# The prevalence and risk factors of preterm small-for-gestational-age infants: a population-based retrospective cohort study in rural Chinese population

**DOI:** 10.1186/s12884-017-1412-7

**Published:** 2017-07-20

**Authors:** Shi Chen, Rong Zhu, Huijuan Zhu, Hongbo Yang, Fengying Gong, Linjie Wang, Yu Jiang, Bill Q. Lian, Chengsheng Yan, Jianqiang Li, Qing Wang, Shi-kun Zhang, Hui Pan

**Affiliations:** 1 Department of Endocrinology, Key Laboratory of Endocrinology of Ministry of Health, Chinese Academy of Medical Sciences & Peking Union Medical College, Peking Union Medical College Hospital, No.1, Shuaifuyuan Road, Beijing, Dongcheng district 100730 China; 2 Intern of medicine, PUMCH, Beijing, 100730 China; 3 School of public health, PUMC, Beijing, 100730 China; 4 University of Massachusetts Medical Center, 55 Lake Ave., North Worcester, MA 01655 USA; 5 Hebei Center for women and children’s health, Shijiazhuang, 050031 China; 6 School of Software Engineering, Beijing University of Technology, Beijing, 100124 China; 7 Tsinghua National Laboratory for Info. Science and Technology, Tsinghua University, Beijing, 100084 China; 8 Research association for women and children’s health, Beijing, 100081 China; 9 Department of Gynaecology and Obsterics, Peking University First Hospital, Beijing, 100034 China

**Keywords:** Preterm delivery, Small for gestational age, Folic acid supplementation, Oral contraceptive

## Abstract

**Background:**

Preterm birth and small for gestational age (SGA) are strong indicators of neonatal adverse outcomes. With the growing importance of preterm SGA infants, we aim to evaluate the prevalence and risk factors for preterm SGA in China.

**Method:**

We analyzed the data of parents and infants from a population-based cohort research of the free National Pre-pregnancy Checkups Project (NPCP) in rural China. Only singleton live births that occurred between 24 weeks +0 days and 36 weeks +6 days of pregnancy were included in this study. SGA was defined as birth weight less than the 10th percentile of the reference birth-weight-for-gestational-age population. A multiple logistic regression model was built using the statistically significant variables from the 371 variables in the questionnaire.

**Results:**

A total of 11,474 singleton, preterm, live-birth infants were included. Of the total infants, 317 (2.77%) were preterm SGA infants. A higher risk of preterm SGA infants was observed among mothers who were on oral contraceptives (OR: 8.162, 95% CI: 1.622–41.072), mothers who had syphilis (OR: 12.800, 95% CI: 1.250–131.041), and mothers with a high eosinophil percentage (OR: 13.292, 95% CI: 1.282–135.796). Maternal intake of folic acid at least 3 months before pregnancy (OR: 0.284, 95% CI:0.124–0.654) and paternal intake of egg and meat (OR: 0.097,95% CI:0.030–0.315) were protective factors. Compared with North China, the incidence of preterm SGA infants was higher in South China.

**Conclusion:**

Preterm SGA infants were associated with both maternal and paternal factors.

## Background

Gestational age and birth weight are two of the most important factors for evaluating the prognosis of infants. Small for gestational age (SGA) infants may show a decrease in their growth due to intrauterine growth restriction. Limitations in fetal growth affect the development of the cardiovascular system or other organs, which can have life-long effects on an individual [1]. Preterm birth is a significant causative factor of infant and child morbidity and mortality. Preterm birth complications are estimated to be the second most common cause of death in children under 5 years old [2]. In addition to its contribution to mortality, preterm birth has lifelong effects, and increased risk of neurodevelopmental disorders and chronic diseases in adulthood [3]. There is a growing consensus on the differentiation of preterm SGA from term SGA infants from both the clinical and research perspectives [4]. In particular, preterm SGA infants have a 10–40 times greater risk of dying in the first month of life than term appropriate for gestational age (AGA) infants [5]. Further, preterm SGA infants have a relatively low body fat percentage and would experience a postnatal catchup growth. Many epidemiological studies have demonstrated that the catch-up growth is associated with cardiovascular diseases, obesity, hypertension, type-2 diabetes, and metabolic syndrome in later life [6]. Few studies have evaluated the risk factors of preterm SGA infants [7, 8]. The purpose of present study is to identify the risk factors of preterm small-for-gestational age infants. The knowledge gained from this study will be crucial in prevention and treatment of preterm SGA.

## Methods

### Subjects

A population-based retrospective cohort study was performed on 248,501 couples and their children who were part of the free National Pre-pregnancy Checkups Project (NPCP) in 220 pilot counties in 30 provinces in China between January 2010 and December 2012. The project was implemented by the Chinese National Health and Family Planning Commission and Ministry of Finance with aim of preventing birth defects in China, it is the largest pregnancy retrospective cohort study of the preconception stage in China. It covered all volunteer couples who planned to conceive within the next 6 months. The clinical data were collected during the preconception medical examination. Information on socioeconomic background, reproductive history and history of illness, lifestyle, and dietary habits was carefully collected through face-to-face interviews by qualified nurses. Physical examinations and biochemical studies were also carried out by medical staff at the same time [9].

SGA was defined by a 1995 WHO expert committee as infants with body weight below the 10th percentile of a birth-weight-for-gestational-age, using the gender-specific reference population with the local growth standards of Li Zhu et al. [10] Zhu’s neonatal growth standards were derived from birth weight data obtained from a nationwide neonatology network of 161,420 live births in China from 2011 to 2014. Preterm SGA infants in our study were defined as infants born small for gestational age between 24 weeks + 0 days and 36 weeks +6 days of gestation.

**Fig. 1:**
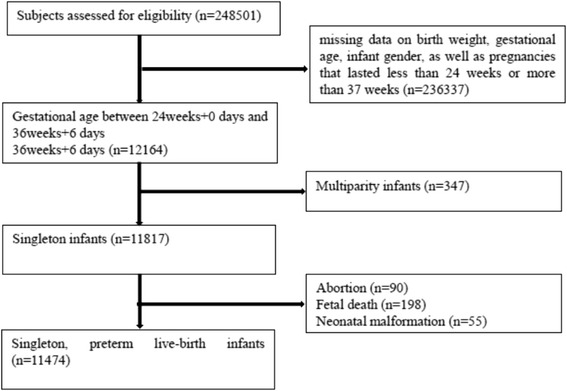
Participant flow chart

The inclusion and exclusion criteria are shown in Fig. 1. A couple and their children was considered as a single subject. We included a total of 11,474 subjects.

### Design and setting

#### Data collection

A structured questionnaire was constructed by well-trained investigators; the questionnaire included 371 variables from the National Free Preconception Health Examination Project [9, 11]. As the adverse effect of preterm large-for-gestational age (LGA) infants is controversial [12, 13], we compared preterm SGA infants with preterm non-SGA infants including the preterm AGA and LGA infants. We divided China into North and South region by the Qinling Mountain-Huaihe River Line and we compared the prevalence of preterm SGA infants in both regions. We also assessed the risk factors of preterm SGA infants.

#### Selection of risk factors

The questionnaire involves 19 aspects including baseline characters of couples, physical examination, laboratory examination family history of couples etc. We chose the variables with a high data integrity over 80%. Among these variables, 38 variables were statistically significant exposures in univariate analysis including education level of the parents, maternal preconception intake of narcotics and paternal second-hand smoking, maternal intake of eggs and meat, the beginning time of maternal intake of folic acid, paternal intake of eggs and meat and paternal intake of vegetables, tense maternal and paternal relationship with relatives and co-workers, paternal exposure to heavy metals, organic solutes and vibrations, maternal syphilis and Candida infection, maternal drug use, pet exposure and influenza virus infection during pregnancy, maternal medical history of diabetes mellitus (DM), maternal medical history of hepatitis B, maternal oral contraceptive use, maternal family history of neonatal death, paternal hepatitis B vaccination and paternal family history of DM, maternal height (meter), maternal weight (kilogram), maternal BMI (kg/m2), maternal red blood cell count (109/L), maternal eosinophil percentage, maternal blood glucose level (mmol/L) and paternal height. It also included the presence of maternal HBe antibodies, maternal rubella virus IgG antibodies, maternal CMV IgG antibodies, maternal toxoplasma IgG antibodies, paternal HBs antibodies. The folic acid supplementation, the preconception habits and socioeconomic status of the parents were based on self-report. The location was classified as North or South region of China by the Qinling Mountain-Huaihe River Line.

### Statistical analysis

All risk factor variables were first examined by univariate analysis to assess the importance of each of them on preterm SGA. We used chi-square test for analysis of categorical variables, and the Mann-Whitney *U* test for analysis of continuous variables with a skewed distribution as all of continuous variables were of skewed distribution in this cohorts examined by Kolmogorov-Smirnov test. The continuous skewed variables were expressed in the form of mid-values (25th percentile, 75th percentile). When a variable was found to be significant at the 0.1 level, it was entered into the multivariate model. Stepwise logistic regression was used to examine the correlation between risk factors and preterm SGA. In logistic regression, *p* < 0.05 was considered statistical significance. The results were presented using the OR and 95% CI values. The analyses were performed with SPSS (version 19.0; SPSS Inc., Chicago, IL, USA).

## Results

### Baseline characteristics of SGA and non-SGA

A total of 248,501 infants were recruited in our database, of which 12,164 were preterm infants (5.63%). The preterm neonatal mortality was 1.69% in our study. The mean weight of 11,474 preterm singleton live-birth infants was 3104.87 ± 636.03 g, 6307 infants of them were male (54.97%). Among them, 317 (2.77%) were preterm singleton live-birth SGA infants. The mean weight of them was 1778.26 ± 438.58 g, 189 of them were male (59.62%). 11,157 of them were preterm singleton live-birth non-SGA infants, the mean weight was 3141.73 ± 595.51 g, 6118 of them were male (54.84%).

### Univariate analysis

The following tables show the risk factors in preterm SGA deliveries. Table 1 indicates that maternal intake of narcotics lead to a higher risk of preterm SGA. Nutrition status is also described in Table 1. Parents who did not consume enough vegetables, eggs or meat were more likely to deliver preterm SGA infants, which reflects the paternal level of essential vitamin and proteins. Starting time of maternal folic acid supplement is described in Table 1. Mothers who used folic acid at least 3 months before last menstrual period (LMP) had a lower risk of giving birth to preterm SGA infants. We also noticed that a total of 3701 women (32.6%) in our study did not take folic acid before or after their pregnancy, even though it is routinely recommended by their health care providers.

**Table 1:** The univariate analysis of risk factors of preterm SGA infants (categorical variables)

Risk factors	Number of SGA	Number of Non-SGA	*P* value
Maternal Education years			
0	2	27	0.000
0–6	19	444	
6–9	181	7743	
9–12	71	1713	
12–16	38	966	
> 16	1	6	
Paternal Education years			
0	1	13	0.008
0–6	13	337	
6–9	189	7464	
9–12	70	1922	
12–16	33	1066	
> 16	0	10	
Maternal intake of narcotics			
Yes	4	29	0.012
No	208	10,782	
Paternal second-hand smoking			
Regular	13	358	0.091
Occasional	90	3732	
No	191	6089	
Maternal intake of eggs and meat			
No	9	155	0.045
Yes	292	10,675	
Maternal intake of vegetable			
No	10	94	0.000
Yes	290	10,737	
Maternal intake of folic acid from at least 3 months before LMP			
Yes	73	3448	0.003
No	241	7584	
Paternal intake of eggs or meat			
No	8	125	0.003
Yes	286	10,073	
Paternal intake of vegetables			
No	5	81	0.094
Yes	289	10,104	
Maternal tense relationship with relatives and co-workers			
No	272	10,098	0.000
Low	29	597	
Moderate	0	143	
High	2	3	
Paternal tense relationship with relatives and co-workers			
No	261	9403	0.075
Low	2	627	
Moderate	4	162	
High	0	5	
Paternal exposure to heavy metals			
Yes	4	28	0.011
No	313	11,489	
Paternal exposure to organic solutes			
Yes	5	528	0.006
No	312	10,989	
Paternal exposure to vibrations			
Yes	4	54	0.076
No	313	11,463	
Maternal syphilis infection			
Yes	4	29	
No	293	10,656	
Maternal Candida infection			
Yes	2	80	0.003
No	269	10,138	
Maternal HBe antibodies			
Positive	31	770	0.024
Negative	255	9855	
Maternal rubella virus IgG antibodies			
Positive	136	4114	0.022
Negative	153	6355	
Maternal CMV IgG antibodies			
Positive	79	2312	0.068
Negative	204	8022	
Maternal toxoplasma IgG antibodies			
Positive	9	148	0.052
Negative	273	10,190	
Paternal HBs antibodies			
Positive	83	2555	0.077
Negative	198	7413	
Maternal medication us after LMP			
Yes	20	274	0.000
No	294	10,758	
Maternal pet exposure after LMP			
Yes	11	171	0.018
No	305	10,942	
Maternal influenza virus infection after LMP			
Yes	9	92	0.002
No	305	11,021	
Maternal medical history of hepatitis B			
Yes	4	51	0.076
No	299	10,811	
Maternal oral contraceptive use			
Yes	4	56	
No	300	10,732	
Maternal family history of neonatal death			
Yes	2	4	0.010
No	300	10,846	
Paternal hepatitis B vaccination			
Yes	66	2980.017	
No	230	7273	
Paternal family history of DM			
Yes	4	47	0.055
No	292	10,716	
Location			
North	60	3671	0.000
South	257	7486	

Paternal exposure to heavy metals, organic solutes and vibrations were associated with a higher incidence of preterm SGA. Parental infections were also identified as important risk factors. Syphilis, Candida infection, rubella virus infection, CMV infection, toxoplasma infection were associated with higher rate of preterm SGA. Hepatitis B is common in China. Positive maternal HBe antibodies is associated with higher prevalence of preterm SGA. Moreover, maternal family history of hepatitis B was associated with higher rate in preterm SGA infants while paternal hepatitis B vaccination was associated with lower rate in preterm SGA infants.

Mothers who were taking medications, came into contact with pets or had influenza virus infection were more likely to have preterm SGA infants. With regard to the medical history of the parents, maternal family history of neonatal death were associated with a higher rate of preterm SGA.

The Qinling Mountain-Huaihe River Line is an important demarcation line of climate, hydrology, and topography in China [14]. The North China has a lower rate of preterm SGA rate (1.61% vs. 3.32%). The mean birth weight of preterm live-birth was 3188.58 ± 650.67 g in North China and 3061.78 ± 636.03 g in South China.

As expected, the parental weight, height and BMI were associated with preterm SGA as shown in Table 2. The median values were used for risk factors that showed skewed distribution. The maternal weight, height, BMI and paternal height were significantly lower and the maternal eosinophil ratio was higher in preterm SGA group.

**Table 2:** The univariate analysis of risk factors of preterm SGA infants (continuous variables)

Risk factors	SGA Median(quartile)	Non-SGA Median(quartile)	*P* value
Maternal age	24.00 (22.00–27.00)	24.00 (22.00–27.50)	0.571
Maternal height (meter)	159.00 (156.00–161.00)	160.00 (156.00–162.00)	0.081
Maternal weight (kilogram)	52.00 (48.00–56.00)	52.00 (49.00–57.00)	0.027
Maternal BMI before LMP (kg/m2)	20.32 (18.89–22.31)	20.70 (19.38–22.38)	0.063
Maternal red blood cell count (10^9^/L)	4.22 (3.90–4.51)	4.13 (3.80–4.48)	0.005
Maternal eosinophil percentage	2.00 (0.73–3.48)	1.10 (0.10–2.50)	0.017
Maternal blood glucose level (mmol/L)	4.90 (4.39–5.50)	4.82 (4.30–5.30)	0.018
Paternal height (meter)	170.00 (168.00–173.25)	171.00 (169.00–175.00)	0.031

### Multivariable analysis

Table 3 shows the results of multiple logistic regression of preterm SGA. Higher risks of preterm SGA infants were observed among women who took oral contraceptives (OR: 8.162, 95% CI: 1.622–41.072), women with higher eosinophil percentage (OR: 1.067, 95% CI: 1.010–1.127) and women with syphilis infection (OR: 13.292, 95% CI: 1.282–135.796). Frequent intake of meat and egg of father (OR: 0.097, 95% CI: 0.030–0.315) was found to be a protective factor for infants. Comparing with women who did not use folic acid or started using folic acid after 3 months before LMP, intake of folic acid from 3 months before LMP (OR: 0.284, 95% CI:0.124–0.654) was also a protective factor for preterm SGA infants. It is well accepted that maternal BMI before LMP is related to the rate of preterm and SGA. So we put the maternal BMI before LMP (OR: 0.945, 95% CI: 0.828–1.709) in the regression model although it was not statistically significant. Moreover, the confidence intervals are wide for some of the factors in the logistic model may due to the small sample size of preterm SGA.

**Table 3:** Multiple logistic regression of preterm SGA infants

Risk factors	B	*P* value	OR	95% C.I. for OR	
				Lower	Upper
Maternal intake of folic acid from at least 3 months before LMP	−1.257	0.003	0.284	0.124	0.654
Maternal oral Contraceptive use	2.100	0.011	8.162	1.622	41.072
Maternal eosinophil percentage	0.064	0.021	1.067	1.010	1.127
Maternal syphilis infection	2.580	0.030	13.191	1.281	135.796
Paternal intake of egg and meat	−2.336	0.000	0.097	0.030	0.315
Maternal BMI before LMP	−0.056	0.403	0.945	0.828	1.709
Constant	1.357	0.401	3.886		

## Discussion

Birth weight and gestational age are considered as strong predictors of short-term and long-term prognosis of infants. Given the growing attention paid to preterm SGA infants, our study attempted to determine the incidence of the preterm SGA infants and the risk factors associated with delivering preterm SGA infants.

A major strength of this study is its large sample size and the large number of variables analyzed. To the best of our knowledge, this is the most extensive multi-center study in China to evaluate the risk factors associated with preterm SGA infants. The large number of variables allows us to analyze more risk factors than previous studies on preterm SGA infants. The effect of paternal factors on preterm SGA infants, for example, the maternal eosinophil percentage has rarely been reported before.

This database has several unique features. Compared with earlier study, the mortality rate of preterm infants in our study (5.63%) was lower than the average rate reported for eastern Asia (7.2% (5.4–9.0)) [15]. With economic growth and improvements in perinatal care, the neonatal mortality rate has decreased by 59.3% from 2000 to 2010 in China [16], which could be due to lower rate of preterm SGA. With regard to the low prevalence of preterm SGA infants in the North China, it could be explained by the significant difference in body weight and height between the Northern and Southern Han Chinese. It also fit the Bergmann’s rule as body mass increases with colder climate [17, 18]. The greater weight and height of parents in the North could explain the lower incidence of preterm SGA in North China.

In our study, we discovered a gender-based difference in the incidence of preterm SGA infants in China; 59.62% of preterm SGA infants were male. It has been reported that boys are more likely to be born before term in a different of populations [19]. A possible explanation is that in preterm infants, the growth-promoting effect of androgen is not obvious. Moreover, the male preterm infants were more likely to meet the preterm SGA criteria, as the weight standard for males is higher than that for females.

### Folic acid

Insufficient periconceptional folic acid intake is associated with a number of birth defects that may also be related to genetic and environmental factors before conception or during early pregnancy [20]. Recent study has shown that supplementation of folic acid could protect against preterm birth. This study also suggests that the duration of folic acid supplementation may be as important as the dose. The risk of spontaneous preterm birth was inversely related to the duration of folic acid supplementation, and was lowest in women who reported using folic acid supplementation for more than a year prior to conception [21]. However, it is controversial whether folic acid supplementation influence the incidence of low birth weight or SGA [21–23]. In our study, taking folic acid supplementation more than 3 months before LMP was associated with a significant reduction in incidence of preterm SGA. As mentioned before, 32.6% of the women in this study did not take folic acid before or during pregnancy, even it is routinely recommended. Considering the large percentage of subjects were from rural areas with relatively poor nutrition status, we think that health care providers in these areas, in particular, should emphasize on folic acid supplementation before pregnancy.

### Oral contraceptive

Oral contraceptives use is one of the most popular reversible methods of contraception. However, the adverse effects of oral contraceptives on fetal development are unclear. Previous studies have reported the association of oral contraceptive use and preterm birth and low birth weight [24, 25]. It should be noted that oral contraceptive use is rare in China compared to developed countries; only 1.31% of women who delivered preterm SGA infants and 0.47% of women who delivered preterm non-SGA infants used oral contraceptives. In contrast, it was reported that oral contraceptive account for 79% of all contraception in America for the same period [26]. Nonetheless, we observed that the use of oral contraceptives was associated with preterm SGA infants. A possible explanation is that increased levels of estrogen at the time of blastocyst implantation may contribute to an increased risk of preterm birth, which has been shown in women undergoing fresh embryo-based transfer for in vitro fertilization [27, 28]. It is undeniable that oral contraceptives have many advantages in birth control and regulating the menstrual cycle, but physicians should be aware of its potential side effects of delivering preterm SGA infants.

### Maternal eosinophil percentage

Eosinophils have been shown to be a significant cellular infiltrate of the placenta and uterus, including the infiltration and degranulation of eosinophils in the cervix of pregnant humans [29]. The roles of eosinophils in preterm delivery or SGA remains unknown. Elevation of the eosinophil level is associated with chronic inflammation or enhanced immune reactions, which may associate with preterm SGA infants. As the eosinophil percentage is not routinely determined in pregnancy, further research needs to be conducted to explore the relationship between the eosinophil percentage and pregnancy.

### Infection of syphilis

Despite being easily detectable and treatable during pregnancy, syphilis remains an important cause of adverse pregnancy outcomes [30]. Syphilis in pregnancy may lead to severely adverse pregnancy outcome such as abortion, prematurity, neonatal death and congenital syphilis in the newborn [31]. In China, the incidence of congenital syphilis has increased at an alarming rate of 71.9% per year from 0.01 to 19.68 cases per 100,000 live births from 1997 to 2005 [32]. A study released in 2013 indicated that total incidence of maternal syphilis in China was estimated as 0.30% (95% CI: 0.28–0.32) [32]. In our study, the incidence of maternal syphilis was 1.35% in women who delivered preterm SGA infants, which was much higher than the incidence in women who delivered preterm non-SGA infants (0.271%). Unless testing and treatment of syphilis during pregnancy are made universally available, over half of the pregnancies in women with syphilis will have adverse outcome [33]. Primary prevention and prenatal care are needed to be addressed to reduce the incidence of syphilis associated preterm SGA infants.

### Nutrition status of father

Our study showed that diet containing egg and meat of the father, which reflected the paternal nutritional status, particularly protein intake, was significantly associated with lower incidence of preterm SGA infants. Animal studies have demonstrated that all stages of gamete maturation and preimplantation embryo development are influenced directly by parental nutrition and hormonal status [34]. Moreover, an animal model showed that the diet during the preconception period and pregnancy of the males and females differentially affects embryonic growth and fatty acid content [35]. Also, there is an animal study showed that paternal nutrition can influence the amount of seminiferous tissue, spermatogenic capacity and spermatogenic efficiency [36]. However, our understanding of the influence of paternal nutritional status on human offspring is still limited.

### Limitation

The primary limitation of our study is that several risk factors such as the beginning time of maternal folic acid intake, the paternal intake of egg and meat, and the use of oral contraceptive were based on self-report of the parents. More quantitative variables are needed in our questionnaire. With the large number of subjects, it is difficult to assure the completeness of data. This study identified several factors that are associated with preterm SGA, due to diverse culture and social economic status of these subjects, some confounding factors might be overlooked.

## Conclusion

Our results show that preterm SGA infants were associated with both maternal and paternal factors. Maternal use of oral contraceptives, maternal syphilis infection, maternal higher eosinophil percentage, maternal folic acid intake less than 3 months before pregnancy and paternal low protein diet were associated with preterm SGA.

## Abbreviations

AGA: Appropriate for gestational age
DM: Diabetes mellitus
LGA: Large for gestational age
LMP: Last menstrual period
SGA: Small for gestational age
